# Impact of intrafraction motion in pancreatic cancer treatments with MR-guided adaptive radiation therapy

**DOI:** 10.3389/fonc.2023.1298099

**Published:** 2023-12-13

**Authors:** Doris N. Rusu, Justine M. Cunningham, Jacob V. Arch, Indrin J. Chetty, Parag J. Parikh, Jennifer L. Dolan

**Affiliations:** ^1^ Department of Radiation Oncology, Wayne State University, Detroit, MI, United States; ^2^ Department of Radiation Oncology, Henry Ford Health System, Detroit, MI, United States; ^3^ Department of Radiation Oncology, Cedars Sinai Medical Center, Los Angeles, CA, United States

**Keywords:** intrafraction motion, gastrointestinal motion, respiratory-gated radiation therapy, stereotactic body radiation therapy (SBRT), MR-linac, MR-guided radiation therapy, online adaptive radiation therapy, pancreatic cancer

## Abstract

**Purpose:**

The total time of radiation treatment delivery for pancreatic cancer patients with daily online adaptive radiation therapy (ART) on an MR-Linac can range from 50 to 90 min. During this period, the target and normal tissues undergo changes due to respiration and physiologic organ motion. We evaluated the dosimetric impact of the intrafraction physiological organ changes.

**Methods:**

Ten locally advanced pancreatic cancer patients were treated with 50 Gy in five fractions with intensity-modulated respiratory-gated radiation therapy on a 0.35-T MR-Linac. Patients received both pre- and post-treatment volumetric MRIs for each fraction. Gastrointestinal organs at risk (GI-OARs) were delineated on the pre-treatment MRI during the online ART process and retrospectively on the post-treatment MRI. The treated dose distribution for each adaptive plan was assessed on the post-treatment anatomy. Prescribed dose volume histogram metrics for the scheduled plan on the pre-treatment anatomy, the adapted plan on the pre-treatment anatomy, and the adapted plan on post-treatment anatomy were compared to the OAR-defined criteria for adaptation: the volume of the GI-OAR receiving greater than 33 Gy (V33Gy) should be ≤1 cubic centimeter.

**Results:**

Across the 50 adapted plans for the 10 patients studied, 70% were adapted to meet the duodenum constraint, 74% for the stomach, 12% for the colon, and 48% for the small bowel. Owing to intrafraction organ motion, at the time of post-treatment imaging, the adaptive criteria were exceeded for the duodenum in 62% of fractions, the stomach in 36%, the colon in 10%, and the small bowel in 48%. Compared to the scheduled plan, the post-treatment plans showed a decrease in the V33Gy, demonstrating the benefit of plan adaptation for 66% of the fractions for the duodenum, 95% for the stomach, 100% for the colon, and 79% for the small bowel.

**Conclusion:**

Post-treatment images demonstrated that over the course of the adaptive plan generation and delivery, the GI-OARs moved from their isotoxic low-dose region and nearer to the dose-escalated high-dose region, exceeding dose-volume constraints. Intrafraction motion can have a significant dosimetric impact; therefore, measures to mitigate this motion are needed. Despite consistent intrafraction motion, plan adaptation still provides a dosimetric benefit.

## 1 Introduction

The superior soft tissue contrast of magnetic resonance imaging (MRI) has long enabled better delineation of targets and normal tissues in the radiotherapy planning workflow (1). Recent significant advances in physics and engineering technology have led to the capability of integrating MRI systems in the radiation treatment room (2–6). It has been argued that such MRI Linear accelerators (MR-Linac) or MR-guided-radiotherapy (MRgRT) machines offer “next generation” image-guided radiotherapy given their ability to provide enhanced soft-tissue, non-ionizing imaging of tumors and surrounding healthy tissues in real time (7–10). MRgRT has now been applied to treatment of tumors in several different anatomic sites (11–24), where it has demonstrated superior imaging of tumors and surrounding healthy tissues and, consequently, reduction of planning margins for facilitating hypo-fractionated RT. Moreover, volumetric MRIs at the time of treatment have permitted workflows to assess and account for daily anatomical variation in the context of online adaptive radiotherapy (ART) (5, 11, 25, 26).

One of the most compelling arguments for MRgRT is for the safe, hypo-fractionated treatment of tumors undergoing motion due to respiration or other physiologic processes during treatment. An example of such an indication is the targeting of locally advanced pancreatic cancer (LAPC) tumors, often impacted by both respiratory and gastrointestinal motion, and limited in visibility using x-ray imaging due to lack of soft-tissue contrast—making a convincing case for MRgRT. Investigators have argued that significant potential exists to improve progression-free survival (PFS) and overall survival (OS) at even higher doses, but that safe delivery of higher doses (>40 Gy) should be accompanied by smaller planning margins and real-time imaging afforded by MRgRT (11, 24, 27–35). Related to MRgRT, a meta-analysis showed that LAPC patients treated with concurrent chemotherapy and radiation therapy at high doses (BED10 >70 Gy) had statistically significant improvement in 2-year OS (49% vs 30%, *p* = 0.03) and trended towards significance for 2-year freedom from local failure (77% vs 57%, *p* = 0.15) compared to standard-dose patients (BED10 ≤70) (33). The treatment was also well tolerated with grade 3+ GI toxicity occurring in 3 out of 20 patients in the standard-dose group and none of the 24 patients in the high-dose arm (33). Online treatment adaptation occurred more frequently in the high dose (83%) vs. the standard dose groups (15%). These promising safety results were confirmed in a large prospective multi-institutional study where treatment of 136 patients to 50 Gy over five fractions resulted in 0 definitely related gastrointestinal toxicities at 90 days (36).

**Figure 1:**
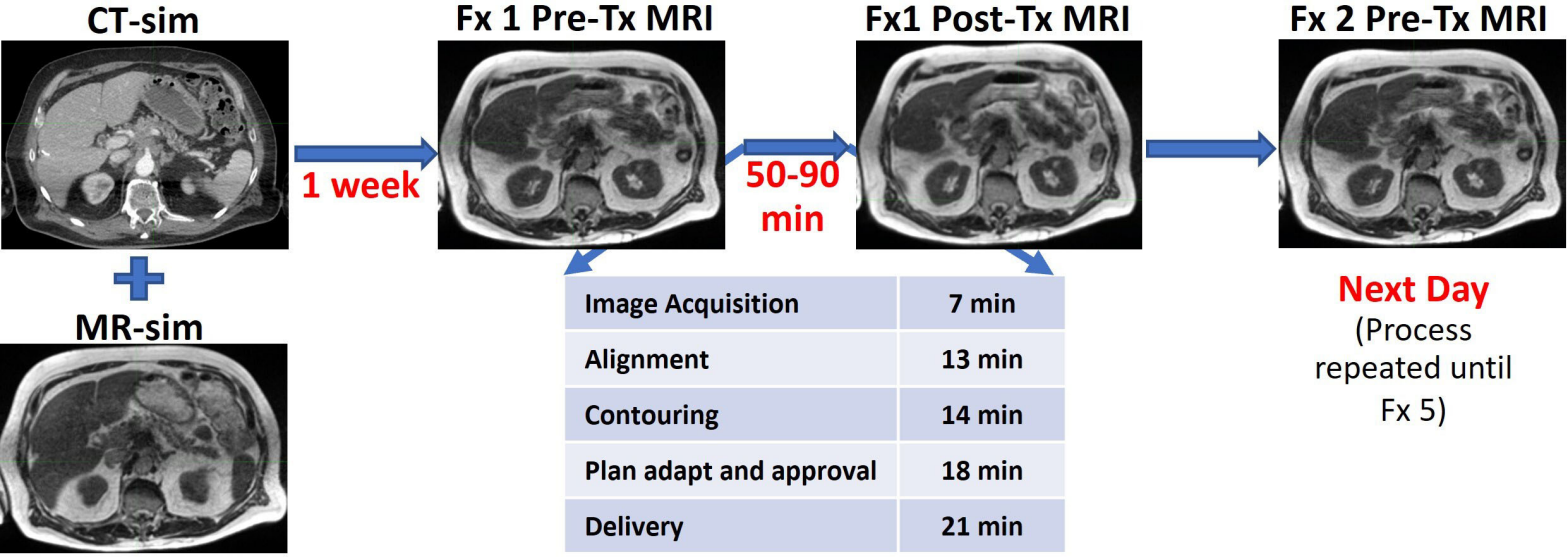
Time frame of MR-guided adaptive radiotherapy as performed at Henry Ford Health. The table shows the average times for each step.

Planning and delivery of online ART involves several procedures, such as segmentation on daily image datasets, plan re-optimization and evaluation, and physics QA, as outlined in **Figure 1**, resulting in overall treatment times for patients with LAPC ranging from 50 to 90 min. Therefore, there can be significant time lapse between the time the patient is first imaged until the time the patient is treated (latent period). It is well known that peristaltic changes of the gastrointestinal tract happen in a stochastic method, but the frequency and severity of changes that could occur during adaptive radiation delivery are not clear. A recent report from Tyagi et al. looked at this for 10 patients treated with MRgRT with abdominal compression during a free breathing treatment, and found that there was a significant amount of patients who had post-treatment imaging that showed dosimetrically significant organ motion for their dosimetry constraints and planning techniques (35). We wanted to investigate the dosimetric impact of intrafraction organ motion in our population, who are treated using breath hold with real-time imaging-based gating during treatment.

## 2 Materials and methods

### 2.1 Patient population and plan generation

The treatment technique for stereotactic MR-guided adaptive radiation therapy (SMART) for pancreas cancer using a low-field MRgRT has been previously described (36). In brief, 10 patients were treated with 50 Gy in five fractions on a 0.35-T MR-Linac (MRIdian, ViewRay Inc, Oakwood Village, OH). A summary of clinical characteristics of this patient cohort can be found in **Table 1**. In short, nine tumors were located in the head of the pancreas, and 1 in the body. Of the 50 treated fractions, 100% of the fractions have one or more organs at risk (OARs) within a 3-mm radius of the clinical target volume (CTV). Patients were instructed not to eat anything 2 h prior to treatment, with no instructions with regard to water and medication consumption. Patients were simulated utilizing a BlueBAG BodyFIX system (BodyFix, Elekta AB, Stockholm, Sweden) in the supine, head-first position with their arms at their sides. Treatment planning CTs were acquired on a big bore radiation therapy CT simulation scanner (Brilliance Big Bore, Philips Health Care, Cleveland, OH), with a 3-mm slice thickness at end exhale with intravenous contrast. Treatment planning MRIs were acquired at end exhale using a true fast imaging and steady precession (TrueFISP) sequence acquired in 17 s with a 45 (left to right) × 45 (anterior to posterior) × 24 (superior to inferior) cm^3^ field of view, a 0.3-cm slice thickness, and a 0.16 × 0.16 cm^2^ in-plane resolution. The CT-simulation image (CT-sim) was rigidly registered to the primary MRI simulation (MR-sim) for target contour delineation; it was then deformably registered to the MR-sim. The MRI was used as the primary image set for treatment planning, with the CT used for assistance in target volume definition and for electron density correction. Plans were generated with 19–22 6X-FFF step-and-shoot static IMRT beams avoiding treatment through the patient arms. Contoured OARs included the duodenum, stomach, colon, small bowel, liver, kidneys, and spinal cord. An integrated Monte Carlo dose optimization and calculation platform was used to produce isotoxic dose distributions by sculpting dose around the limiting gastrointestinal organs at risk (GI-OARs) while escalating dose to the target volumes (37).

**Table 1:** Patient characteristics.

Number of Patients	10
Gender (N)	
**Female**	3
**Male**	7
Pancreas Tumor Location	
**Head**	9
**Body**	1
**Tail**	0
**CTV Size** (cc, median, range)	86 (36-145)
OARS within 3 mm of CTV (% of fractions)	
**Yes**	100
**No**	0

### 2.2 Online adaptive radiation therapy treatment workflow

Treatment localization was performed at end exhale utilizing the same volumetric MRI parameters from simulation. Three-dimensional couch translations were applied based on a target-focused soft-tissue rigid registration. The GI-OARs (duodenum, stomach, colon, and small bowel) were manually segmented and approved by the attending physician. Optimization structures (e.g., CTV_OPT) were created by cropping the target volumes 3 mm away from the GI-OARs. Plans were then reoptimized to escalate dose to these structures while prioritizing GI-OAR sparing. Adaptation was prompted when the scheduled plan OAR prescription constraints were violated or a 10% increase in planning target volume coverage was feasible. The OAR-defined criteria for plan adaptation were that the volume of the GI-OAR receiving greater than 33 Gy (V33Gy) should be kept to less than or equal to 1 cubic centimeter (cc). The adaptation was done while generally maintaining target volume coverage. A summary of target volume coverage (volume that received 100% of the prescription) for the scheduled and adapted plans can be found in the **Supplementary Material**.

### 2.3 Image guidance

During treatment delivery, real-time intrafraction gating was performed utilizing a single sagittal plane MR-Cine image. Cine images were acquired at four frames per second with an in-plane resolution of 0.35 cm × 0.35 cm and a slice thickness of 0.7 cm. The two gating requirements include the gating margin to generate the gating boundary and percentage of the structure allowed outside the gating boundary. The gated beam was triggered on when the tracked tumor volume was within a boundary expansion equivalent to the clinical target volume to planning target volume margin of 0.3 cm and with a 5% tracking volume excursion allowance.

### 2.4 Post-treatment imaging and offline evaluation

Patients underwent a post-treatment volumetric MRI at end exhale to enable the assessment of the dosimetric impact of gastrointestinal anatomical changes during the described latency period. Under an IRB-approved retrospective study, post-treatment MRIs were rigidly registered to pre-treatment MRIs focusing on the alignment of the target volume. This registration was done to adjust for differences in respiratory motion between the pre- and post-treatment breath hold. Since treatment was delivered with real-time imaging of the CTV, and shifts were made during treatment based on CTV movement, this step permits the assumption that any OAR changes seen will be due to peristalsis alone. GI-OAR structures were retrospectively delineated on the post-treatment MRI by a therapist and verified by a physician. The treated dose distribution for each adaptive plan was then overlaid and assessed on the post-treatment anatomy. Prescribed dose volume histogram (DVH) metrics for the (1) scheduled treatment plan on the pre-treatment anatomy, (2) adapted treatment plan on the pre-treatment anatomy, and (3) adapted treatment plan on the post-treatment anatomy were compared to the OAR-defined criteria for plan adaptation: the V33Gy ≤ 1 cc.

To understand the dosimetric impact of intrafraction motion, the V33Gy of the GI-OARs were tabulated for each fraction of the 10 patients at the three time points described above; the full dataset of V33Gy values for each patient can be found in the **Supplementary Material**. The percentage of the 50 treated fractions that exceeded the 1 cc constraint was calculated for each structure; this was determined for both scheduled and post-treatment plans. For each patient, the V33Gy of each GI-OAR was averaged across the five treated fractions for the scheduled and post-treatment plans.

The percentage of fractions that had a greater V33Gy at post-treatment than the adapted plan at pre-treatment were tabulated. To study the benefit of plan adaptation despite intrafraction motion, the number of fractions that had a smaller V33Gy at post-treatment as opposed to the scheduled plan was tabulated. Then, the percentage of fractions that had smaller V33Gy than scheduled was calculated out of the number of fractions in which each OAR initially exceeded the constraint. For example, the duodenum had V33Gy > 1 cc in 35 fractions; of these, 23 fractions had a smaller V33Gy in the post-treatment evaluation; therefore, 66% of fractions were still less than scheduled.

## 3 Results

### 3.1 Post-treatment evaluation

**Figure 2:**
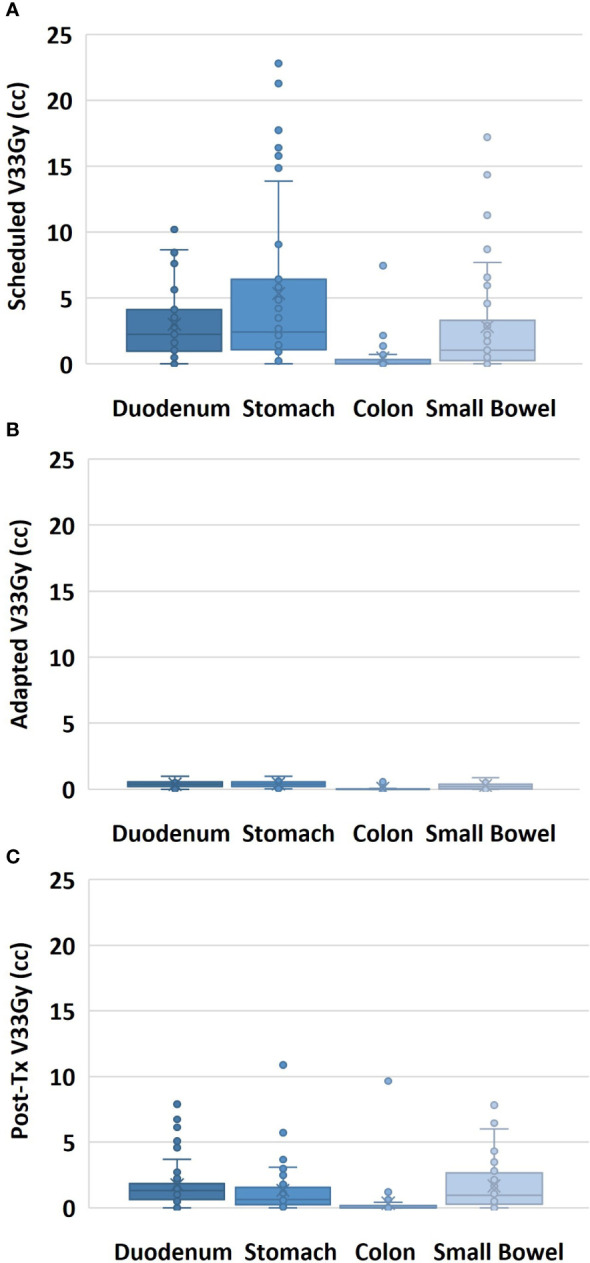
Volume of the GI-OAR within a 3-cm ring of the target volume receiving 33 Gy or more of dose, shown for all fractions for the scheduled plan on pre-treatment anatomy **(A)**, for the adapted plan on pre-treatment anatomy **(B)**, and for the adapted plan on post-treatment anatomy **(C)**.

Of the 50 treated fractions, 100% of the scheduled treatment plans were adapted. The GI-OARs exceeded the adaptive criteria for the duodenum in 70% of fractions, the stomach in 74%, the colon in 12%, and the small bowel in 48%. After treatment plan adaptation, the V33Gy constraint was met by all GI-OARs for all fractions. Analyzing the adapted plan on the post-treatment anatomy, the V33Gy constraint was exceeded for the duodenum in 62% of fractions, the stomach in 36%, the colon in 10%, and the small bowel in 48%. The distribution of the V33Gy DVH metrics for all fractions at the scheduled, adapted, and post-treatment time points can be seen in **Figure 2**.

**Figure 3:**
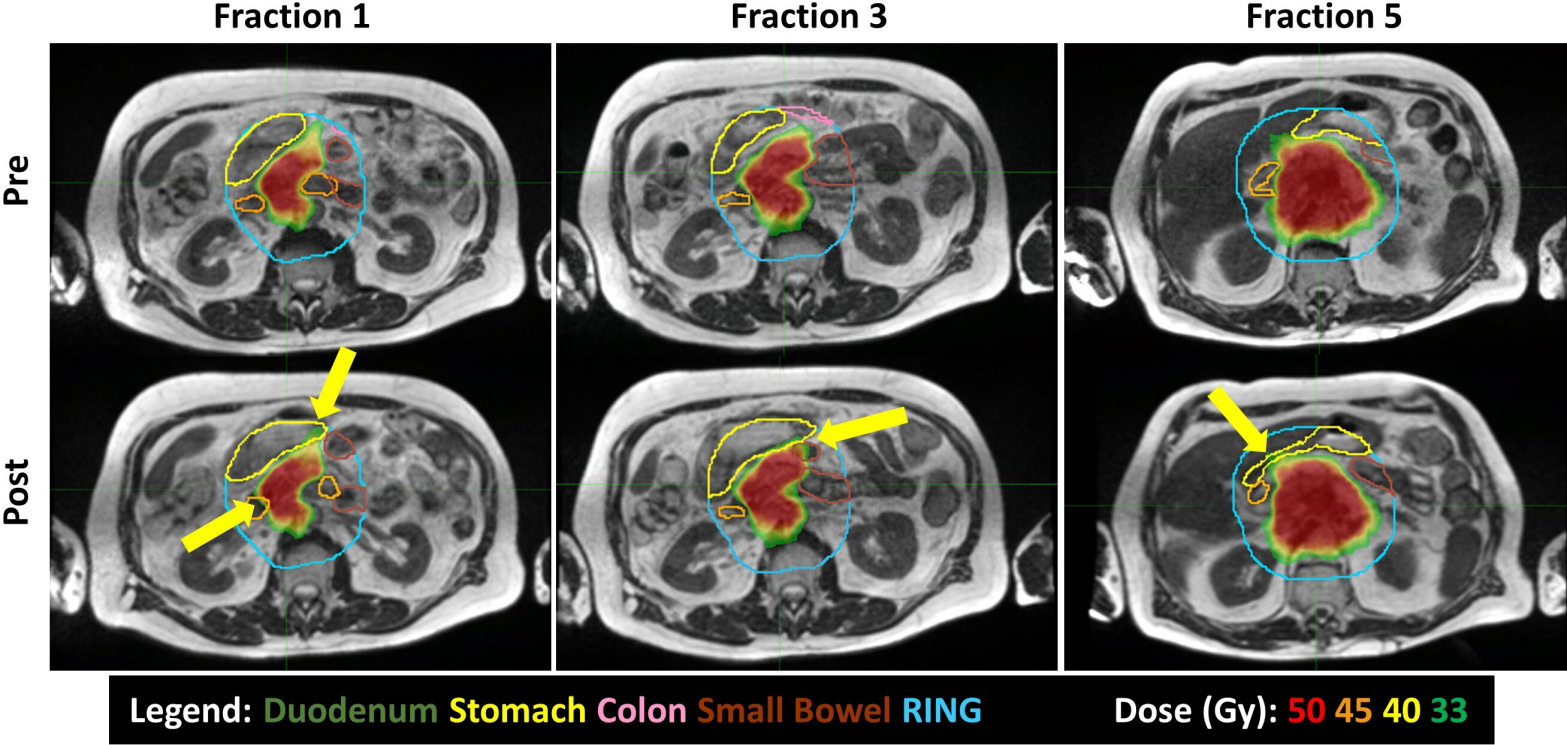
Intrafraction motion seen between pre-treatment (top) and post-treatment (bottom) MRIs, over the course of treatment (Fractions 1, 3, and 5). The duodenum is orange; stomach, yellow; colon, pink; and small bowel, brown. Yellow arrows highlight 33 Gy or higher spilling into the GI OARs.

When averaging the scheduled plan V33Gy for each GI-OAR across a patient’s five-fraction course: nine patients exceeded the duodenum constraint, nine patients exceeded the stomach, two patients exceeded the colon, and five patients exceeded the small bowel constraint. When averaging the post-treatment evaluation plan V33Gy for each GI-OAR across a patient’s five-fraction course: eight patients exceeded the duodenum, five patients exceeded the stomach, one patient exceeded the colon, and five patients exceeded the small bowel. These results are tabulated in **Table 2**. Post-treatment images demonstrated that during a single treatment fraction, over the duration of the adaptive plan generation and treatment delivery, the GI-OARs tended to move from their modulated low-dose regions established during the re-optimization and therefore into high-dose or dose gradient regions. **Figure 3** visually represents one patient’s pre- and post-treatment anatomy and isotoxic dose distribution at different time points in their treatment (fractions 1, 3, and 5).

**Table 2:** Comparison of median and range of V33Gy for all fractions and all patients for each GI-OAR, for the scheduled, adapted, and posttreatment plans. Comparison of the number of patients, out of 10, with average V33Gy (over five fractions) for each GI-OAR that exceeded the constraint, for predicted and post-treatment plans.

Organ at risk	Median V33Gy (cc) (range) Constraint: V33Gy ≤ 1.0 cc			No. of patients on average exceeding constraint N=10	
	Scheduled	Adapted	Post-Tx	Scheduled	Post-Tx
**Duodenum**	2.1 (0–10.3)	0.4 (0–.9)	1.3 (0–7.9)	9	8
**Stomach**	2.4 (0–22.8)	0.4 (0–1.0)	0.6 (0–10.9)	9	5
**Colon**	0.0 (0–7.5)	0.0 (0–1.0)	0.0 (0–9.7)	2	1
**Small** b**owel**	0.7 (0–17.2)	0.3 (0–0.9)	1.0 (0–7.8)	5	5

### 3.2 Benefit of adaptation

**Figure 4:**
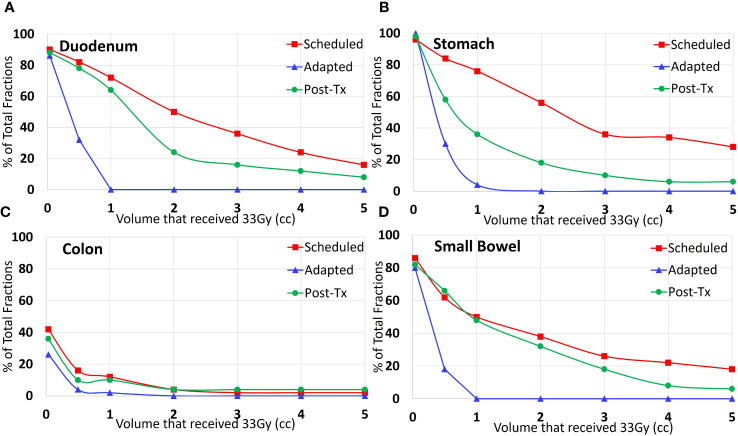
Percent of all treated fractions that received 33 Gy or more to the specified volume of each organ: **(A)** duodenum, **(B)** stomach, **(C)** colon, and **(D)** small bowel. The red line (squares) shows the performance of the scheduled plan on the pre-treatment anatomy; the blue line (triangles) shows the adapted plan optimized based on the pre-treatment anatomy, and the green (circles) shows the adapted plan on the post-treatment anatomy.

The post-treatment evaluation plans showed an increase in the V33Gy value relative to the adapted plan in 94% of the fractions for the duodenum, 68% for the stomach, and 86% for both the colon and small bowel. The post-treatment evaluation plans showed a decrease in the V33Gy value relative to the scheduled plan in 66% of the fractions for the duodenum, 95% for the stomach, 100% for the colon, and 79% for the small bowel. **Table 2** includes the median and range doses for the GI-OARs of all patients and fractions. While the V33Gy for the post-treatment evaluation tended to remain below the V33Gy of the initial scheduled plan, the percentage of fractions that exceed the constraint at the end of treatment is still meaningful. To better understand the benefits of plan adaptation, the performance of the scheduled, adapted pre-treatment, and post-treatment plans were compared at various V33Gy cutoff points. It is important to note that the adapted plans were re-optimized to meet the 1 cc constraint, and this analysis does not consider the performance of plans that have been optimized to meet various V33Gy constraints. For each OAR and each evaluation time point, the percentage of fractions that had a V33Gy of at least 0.035, 0.5, 1, 2, 3, 4, and 5 cc were tabulated. For example, the percentage of fractions that had a V33Gy of 2 cc or more was calculated for each OAR for the scheduled, adapted, and post-treatment plans. The results are visually represented in **Figure 4**.

## 4 Discussion

This is the first study to report on the impact of intrafraction motion in the respiratory-gated breath-hold treatment setting for patients with LAPC being treated with SBRT (50 Gy in 5 fractions) and online ART on a 0.35-T MRgRT system. We found that intrafraction gastrointestinal motion causes violations of dose volume constraints in most fractions, signaling that additional intrafraction motion management is necessary to fully realize the potential of MR-guided online ART. The impact of gastrointestinal motion occurs on a slower and less predictable time scale than respiratory motion. While MRgRT has developed solutions for respiratory motion management, further tools are necessary to minimize the impact of other sources of intrafraction motion, such as gastrointestinal motion. Artificial intelligence (AI) has the potential to bridge this gap in the management of intrafraction motion. Primarily, AI tools can speed up the plan adaptation process, decreasing the time the GI-OARs have to move out of their low-dose regions. AI could also be used during treatment delivery to adapt treatment plans in real time by predicting/anticipating future OAR motion. This application has been broached by Buchanan et al. (38). The data collected within this study can be a useful launching pad for the development of such AI tools.

Within this study, we found that there is a dosimetric difference between the adapted dose to the GI-OARs and the post-treatment dose to the GI-OARs. The post-treatment imaging showed that organs exceeded the dose volume constraints, with consistent increased V33Gy values when compared with the adapted plan. The number of patients with GI-OAR V33Gy averages that exceeded the “criteria to adapt” constraints was similar between the scheduled and post-treatment plans, as seen in **Table 2**. The wide range of V33Gy values within **Table 2** corresponded to two patients in particular, which had associated large deviations within the scheduled and post-treatment plans; this would indicate that these patients had generally larger OAR variations when compared to the rest of the cohort. A more detailed per-patient comparison of V33Gy for scheduled versus post-treatment anatomy for each OAR can be found in the **Supplementary Material**. However, the similar median values do prompt concerns over the benefit of plan adaptation. Therefore, the post-treatment dose-volume results were compared with the scheduled plan in the absence of adaptation, and the post-treatment V33Gy was found to be smaller for most fractions. This can be seen in **Figures 2**, **4**, where the post-treatment V33Gy is less than the scheduled, especially for the stomach and duodenum. In **Figure 4**, the scheduled plan consistently had a larger percentage of fractions that had at least 33 Gy at each volume (from 0.035 cc to 5 cc). Although the post-treatment values at 1 cc receiving 33 Gy were close to the scheduled, the post-treatment values were significantly lower at absolute volume constraints greater than 1 cc. This indicates that the adapted plans gave a dose of 33 Gy to less OAR volume overall as compared to the initial plans; therefore, plan adaptation is meaningful. This could be an indicator of why MR-guided ART has demonstrated success in GI-OAR organ sparing in the SMART trial (36).

These findings align with the findings of Tyagi et al. (35) who studied intrafraction motion for 10 LAPC patients treated with daily, online ART on a 1.5-T MR-Linac (Unity, Elekta AB, Stockholm, Sweden). Tyagi et al. performed imaging prior to plan adaptation, immediately before treatment, and after treatment. Patients were treated with abdominal compression in a free-breathing state, with no beam gating. The median total treatment time per fraction was 75 min. They used GI-OAR constraints of D0.035cc ≤ 33 Gy and D5cc ≤ 25 Gy, with a planning risk volume constraint of D2cc ≤ 33 Gy. The planning risk volume was a 1-mm expansion of the OAR. Plans were optimized to meet all OAR constraints while maximizing target coverage. Plans were verified immediately before treatment, and in eight fractions, the plans were re-adapted at this time point to account for significant volume changes.

Tyagi et al. found that intrafraction motion is critical and varies greatly between patients, which can result in OARs moving into high-dose areas. Contours were adjusted and dose was recalculated on verification and post-treatment images prior to the next fraction to allow for discussion with the physician and modification of plan of action. This resulted in improved performance in later fractions. The percentage of fractions that exceeded their D0.035cc dose constraints for stomach_duodneum and small bowel, at verification and post-treatment, ranged from 42% to 52% and from 52% to 54%, respectively (35). This is comparable to our finding that showed post-treatment metrics exceeding our V33Gy constraint in 48%–62% of fractions for the duodenum, stomach, and small bowel. For comparison, they retrospectively evaluated their dosimetric data with the V33Gy < 1 cc constraint, and their results showed that ~10% of their fractions did not meet the V33Gy < 1 cc constraint (as opposed to 74% in our findings). Adapting their plans to meet more stringent constraints (D0.035cc ≤ 33 Gy), re-planning at the verification time point and implementing the inter-fractional feedback loop are probable factors for the difference in results.

Limitations to this study include that the images and data collected were performed prior to a system upgrade designed around patient throughput, now reducing treatment time to less than 60 min for a majority of patients. Further limitations include the small sample size and the lack of breath-hold repeatability without user and patient feedback for the pre- and post-treatment MRIs. Although the rigid registration between pre- and post-treatment images was done to account for these changes, it may still introduce uncertainty. Additionally, as the pre-treatment contours were developed as part of the patient’s treatment workflow, interobserver contouring variations are present. Another limitation is that the dose was analyzed at two time points, demonstrating a worst-case scenario of assuming the anatomy was at its final position for the entirety of treatment, as opposed to actively deforming and accumulating dose with actively changing OARs during treatment. A larger sample size could be used to achieve more conclusive results. Further developments of accurate, real-time dose accumulation would be needed to form a complete understanding of the dosimetric impact of intrafraction motion. Real-time dose accumulation and AI-driven real-time plan adaptation would be a promising combination to manage intrafraction motion.

## 5 Conclusion

In summary, pre-treatment and post-treatment GI-OAR contours were compared to quantitatively determine the adherence of the dynamic abdominal anatomy to the prescribed constraints over the duration of a single treated fraction. In most cases, one or more of the GI-OARs moved from its designated low-dose region to a high-dose region and resulted in DVH metrics that would trigger a re-optimization in the adaptive planning workflow. While the V33Gy value did increase in the majority of the post-treatment evaluation plans, especially for the duodenum and small bowel, it remained less than the scheduled plan value that triggered the adaptive workflow in the better part of the adapted fractions. We found that intrafraction motion can have a significant dosimetric impact and measures to mitigate intrafraction motion are needed. However, despite consistent intrafraction motion, we found that plan adaptation still provided a dosimetric benefit to our locally advanced pancreatic cancer patient population.

## Data availability statement

The raw data supporting the conclusions of this article will be made available by the authors, without undue reservation.

## Ethics statement

The studies involving humans were approved by Henry Ford Health Institutional Review Board. The studies were conducted in accordance with the local legislation and institutional requirements. The ethics committee/institutional review board waived the requirement of written informed consent for participation from the participants or the participants’ legal guardians/next of kin because it was a retrospective chart study.

## Author contributions

DR: Data curation, Formal analysis, Investigation, Visualization, Writing – original draft, Writing – review & editing. JC: Conceptualization, Data curation, Formal analysis, Investigation, Methodology, Writing – original draft, Writing – review & editing. JA: Data curation, Investigation, Methodology, Writing – review & editing. IC: Resources, Writing – original draft, Writing – review & editing. PP: Conceptualization, Funding acquisition, Investigation, Methodology, Project administration, Writing – review & editing. JD: Conceptualization, Data curation, Formal analysis, Funding acquisition, Investigation, Methodology, Project administration, Supervision, Writing – review & editing.
