# Supplementary material for: Impact of intrafraction motion in pancreatic cancer treatments with MR-guided adaptive radiation therapy
Source: Front Oncol. 2023 Dec 13;13:1298099. doi: 10.3389/fonc.2023.1298099 (PMC10756668; doi:10.3389/fonc.2023.1298099)

Table 1a: Volume (cc) of the duodenum and stomach that received 33 Gy (cc), for each fraction of each patient.

| **Patient Information** | | **Duodenum** | | | **Stomach** | | |
| --- | --- | --- | --- | --- | --- | --- | --- |
| Patient # | Fraction | Scheduled | Adapted | Post_Tx | Scheduled | Adapted | Post_Tx |
| 1 | 1 | 5.91 | 0.71 | 1.20 | 2.95 | 0.15 | 0.25 |
|  | 2 | 0.51 | 0.47 | 0.44 | 0.53 | 0.22 | 0.66 |
|  | 3 | 1.89 | 0.28 | 1.71 | 0.46 | 0.12 | 0.06 |
|  | 4 | 1.60 | 0.29 | 0.34 | 5.82 | 0.28 | 0.26 |
|  | 5 | 2.54 | 0.45 | 1.57 | 4.87 | 0.60 | 0.21 |
| 2 | 1 | 3.90 | 0.43 | 4.58 | 22.81 | 0.43 | 2.99 |
|  | 2 | 3.53 | 0.47 | 1.37 | 2.42 | 0.23 | 1.29 |
|  | 3 | 0.84 | 0.84 | 2.71 | 1.77 | 0.41 | 3.67 |
|  | 4 | 4.44 | 0.57 | 1.00 | 6.43 | 0.98 | 0.66 |
|  | 5 | 2.48 | 0.59 | 0.47 | 1.34 | 0.47 | 1.59 |
| 3 | 1 | 2.54 | 0.21 | 1.49 | 2.44 | 0.14 | 0.92 |
|  | 2 | 1.60 | 0.23 | 0.79 | 3.49 | 0.49 | 1.04 |
|  | 3 | 2.02 | 0.51 | 4.96 | 2.18 | 0.42 | 2.10 |
|  | 4 | 3.73 | 0.56 | 2.86 | 0.61 | 0.23 | 1.79 |
|  | 5 | 4.13 | 0.38 | 1.51 | 1.39 | 0.86 | 0.73 |
| 4 | 1 | 4.57 | 0.37 | 1.42 | 2.24 | 0.28 | 1.52 |
|  | 2 | 5.97 | 0.85 | 1.70 | 0.58 | 0.34 | 0.31 |
|  | 3 | 0.66 | 0.66 | 1.19 | 1.43 | 0.15 | 0.69 |
|  | 4 | 0.97 | 0.97 | 1.44 | 0.92 | 0.07 | 0.26 |
|  | 5 | 1.60 | 0.78 | 7.90 | 1.09 | 0.10 | 0.24 |
| 5 | 1 | 0.00 | 0.00 | 0.00 | 2.41 | 0.08 | 0.22 |
|  | 2 | 0.00 | 0.00 | 0.00 | 1.18 | 0.04 | 0.62 |
|  | 3 | 0.00 | 0.00 | 0.00 | 0.36 | 0.50 | 3.07 |
|  | 4 | 0.00 | 0.00 | 0.00 | 2.68 | 0.05 | 1.04 |
|  | 5 | 0.00 | 0.00 | 0.00 | 9.15 | 0.40 | 0.60 |
| 6 | 1 | 0.49 | 0.02 | 6.73 | 9.07 | 1.00 | 2.49 |
|  | 2 | 10.31 | 0.51 | 1.30 | 2.36 | 0.24 | 0.47 |
|  | 3 | 10.21 | 0.35 | 0.98 | 1.89 | 1.08 | 1.34 |
|  | 4 | 7.80 | 0.71 | 1.47 | 17.75 | 0.97 | 2.58 |
|  | 5 | 4.58 | 0.72 | 6.13 | 0.22 | 0.42 | 5.72 |
| 7 | 1 | 8.45 | 0.56 | 1.63 | 0.42 | 0.29 | 0.41 |
|  | 2 | 5.63 | 0.63 | 5.09 | 2.52 | 0.73 | 0.22 |
|  | 3 | 2.82 | 0.28 | 1.55 | 2.78 | 0.39 | 0.21 |
|  | 4 | 1.04 | 0.38 | 0.65 | 4.22 | 0.72 | 0.12 |
|  | 5 | 1.76 | 0.48 | 2.22 | 4.88 | 0.65 | 0.00 |
| 8 | 1 | 1.54 | 0.26 | 1.28 | 0.02 | 0.25 | 0.62 |
|  | 2 | 1.28 | 0.28 | 0.33 | 1.03 | 0.41 | 0.05 |
|  | 3 | 1.50 | 0.21 | 1.08 | 0.02 | 0.11 | 0.94 |
|  | 4 | 1.49 | 0.25 | 1.72 | 1.12 | 0.30 | 0.29 |
|  | 5 | 3.23 | 0.68 | 0.71 | 0.36 | 0.14 | 0.18 |
| 9 | 1 | 1.47 | 0.23 | 3.06 | 16.41 | 0.86 | 1.14 |
|  | 2 | 8.65 | 0.42 | 0.78 | 5.90 | 0.99 | 1.11 |
|  | 3 | 2.92 | 0.37 | 0.83 | 18.16 | 0.53 | 0.22 |
|  | 4 | 0.96 | 0.16 | 3.69 | 1.03 | 0.53 | 0.61 |
|  | 5 | 0.28 | 0.28 | 1.65 | 0.63 | 0.63 | 0.57 |
| 10 | 1 | 2.24 | 0.21 | 1.19 | 14.88 | 0.41 | 5.88 |
|  | 2 | 1.68 | 0.06 | 2.50 | 15.78 | 0.29 | 0.12 |
|  | 3 | 7.62 | 0.06 | 0.36 | 13.89 | 0.29 | 10.87 |
|  | 4 | 3.84 | 0.02 | 0.50 | 21.29 | 0.43 | 0.27 |
|  | 5 | 3.22 | 0.04 | 0.00 | 15.09 | 0.18 | 0.44 |

Table 1b: Volume (cc) of the colon and small bowel that received 33 Gy (cc), for each fraction of each patient.

| **Patient Information** | | **Colon** | | | **Small Bowel** | | |
| --- | --- | --- | --- | --- | --- | --- | --- |
| Patient # | Fraction | Scheduled | Adapted | Post_Tx | Scheduled | Adapted | Post_Tx |
| 1 | 1 | 0.36 | 0.00 | 0.01 | 5.95 | 0.51 | 2.13 |
|  | 2 | 0.03 | 0.00 | 0.17 | 4.76 | 0.31 | 0.93 |
|  | 3 | 1.12 | 0.05 | 0.00 | 6.77 | 0.42 | 6.45 |
|  | 4 | 0.02 | 0.23 | 0.23 | 8.69 | 0.89 | 1.64 |
|  | 5 | 0.20 | 0.03 | 0.05 | 0.65 | 0.15 | 7.83 |
| 2 | 1 | 0.00 | 0.00 | 0.00 | 11.28 | 0.84 | 2.99 |
|  | 2 | 0.09 | 0.00 | 0.00 | 0.07 | 0.31 | 4.31 |
|  | 3 | 0.72 | 0.04 | 0.00 | 0.71 | 0.38 | 6.02 |
|  | 4 | 0.00 | 0.00 | 0.00 | 1.49 | 0.89 | 3.16 |
|  | 5 | 0.03 | 0.00 | 0.00 | 3.30 | 0.40 | 2.46 |
| 3 | 1 | 0.15 | 0.06 | 0.00 | 2.11 | 0.72 | 2.60 |
|  | 2 | 0.00 | 0.00 | 0.00 | 0.73 | 0.04 | 0.03 |
|  | 3 | 0.00 | 0.00 | 0.00 | 1.04 | 0.04 | 1.93 |
|  | 4 | 0.00 | 0.00 | 0.00 | 3.19 | 0.88 | 1.26 |
|  | 5 | 0.00 | 0.00 | 0.00 | 2.88 | 0.51 | 0.54 |
| 4 | 1 | 0.41 | 0.02 | 0.18 | 1.33 | 0.42 | 0.01 |
|  | 2 | 0.00 | 0.18 | 1.74 | 0.49 | 0.21 | 2.98 |
|  | 3 | 0.00 | 0.19 | 0.00 | 0.58 | 0.33 | 0.21 |
|  | 4 | 0.00 | 0.00 | 0.16 | 0.10 | 0.08 | 0.50 |
|  | 5 | 0.03 | 0.00 | 0.00 | 0.29 | 0.26 | 0.38 |
| 5 | 1 | 0.00 | 0.00 | 0.00 | 0.00 | 0.00 | 0.00 |
|  | 2 | 0.00 | 0.00 | 0.00 | 0.00 | 0.00 | 0.00 |
|  | 3 | 0.00 | 0.00 | 0.03 | 0.00 | 0.00 | 0.00 |
|  | 4 | 0.00 | 0.00 | 0.00 | 0.00 | 0.00 | 0.00 |
|  | 5 | 0.00 | 0.00 | 0.00 | 0.25 | 0.58 | 0.00 |
| 6 | 1 | 0.16 | 0.00 | 0.00 | 6.57 | 0.31 | 1.96 |
|  | 2 | 0.68 | 0.01 | 1.19 | 11.66 | 0.35 | 3.27 |
|  | 3 | 0.25 | 0.04 | 9.65 | 2.21 | 0.13 | 2.80 |
|  | 4 | 0.22 | 0.01 | 0.37 | 14.35 | 0.21 | 3.78 |
|  | 5 | 7.45 | 0.12 | 0.35 | 17.22 | 0.09 | 2.10 |
| 7 | 1 | 1.35 | 1.00 | 0.10 | 2.28 | 0.01 | 3.02 |
|  | 2 | 1.55 | 0.55 | 0.07 | 0.00 | 0.00 | 0.45 |
|  | 3 | 1.40 | 0.32 | 0.40 | 2.21 | 0.29 | 0.01 |
|  | 4 | 0.31 | 0.01 | 0.01 | 0.17 | 0.00 | 3.48 |
|  | 5 | 2.16 | 0.11 | 1.06 | 0.26 | 0.05 | 1.25 |
| 8 | 1 | 0.43 | 0.32 | 0.47 | 0.05 | 0.00 | 0.97 |
|  | 2 | 0.27 | 0.03 | 0.46 | 0.28 | 0.00 | 0.44 |
|  | 3 | 0.47 | 0.00 | 0.00 | 2.20 | 0.04 | 0.70 |
|  | 4 | 0.06 | 0.02 | 0.05 | 4.59 | 0.17 | 1.84 |
|  | 5 | 0.00 | 0.00 | 0.00 | 7.69 | 0.28 | 0.33 |
| 9 | 1 | 0.00 | 0.00 | 0.00 | 0.14 | 0.16 | 1.78 |
|  | 2 | 0.00 | 0.00 | 0.00 | 0.44 | 0.19 | 0.66 |
|  | 3 | 0.00 | 0.03 | 0.00 | 0.25 | 0.01 | 0.25 |
|  | 4 | 0.00 | 0.00 | 0.00 | 0.15 | 0.06 | 0.80 |
|  | 5 | 0.00 | 0.00 | 0.00 | 0.50 | 0.18 | 0.16 |
| 10 | 1 | 0.00 | 0.00 | 0.00 | 0.67 | 0.38 | 0.80 |
|  | 2 | 0.00 | 0.00 | 0.00 | 1.05 | 0.29 | 1.06 |
|  | 3 | 0.00 | 0.00 | 0.00 | 0.41 | 0.29 | 0.78 |
|  | 4 | 0.00 | 0.00 | 0.00 | 0.55 | 0.23 | 0.29 |
|  | 5 | 0.24 | 0.00 | 0.00 | 1.71 | 0.56 | 0.03 |

Table 2: Percent of target volume that received 50 Gy, for clinical target volume (CTV), planning target volume (CTV) and associated optimization structures (CTV_OPT, PTV_OPT), for all fractions of all patients.

|  | | **CTV** | | **CTV_OPT** | | **PTV** | | **PTV_OPT** | |
| --- | --- | --- | --- | --- | --- | --- | --- | --- | --- |
| Patient | Fraction | Scheduled | Adapted | Scheduled | Adapted | Scheduled | Adapted | Scheduled | Adapted |
| 1 | 1 | 78.51 | 66.14 | 87.16 | 79.60 | 62.69 | 49.26 | 77.62 | 66.57 |
|  | 2 | 64.95 | 69.29 | 72.22 | 78.34 | 49.23 | 54.61 | 62.56 | 70.77 |
|  | 3 | 62.77 | 68.45 | 67.93 | 78.96 | 48.64 | 52.96 | 59.40 | 69.44 |
|  | 4 | 65.84 | 63.07 | 73.96 | 74.87 | 51.46 | 47.84 | 66.75 | 66.00 |
|  | 5 | 67.09 | 69.54 | 75.41 | 80.34 | 52.33 | 54.49 | 65.69 | 70.55 |
| 2 | 1 | 93.30 | 79.90 | 95.08 | 86.09 | 79.78 | 67.30 | 85.58 | 76.59 |
|  | 2 | 79.29 | 78.29 | 82.88 | 82.42 | 67.23 | 64.18 | 73.81 | 70.90 |
|  | 3 | 77.81 | 80.24 | 83.84 | 86.39 | 65.84 | 67.70 | 74.88 | 76.92 |
|  | 4 | 80.38 | 76.44 | 83.99 | 82.18 | 67.77 | 63.35 | 74.64 | 71.53 |
|  | 5 | 77.34 | 83.79 | 78.90 | 85.88 | 65.30 | 70.10 | 69.33 | 74.78 |
| 3 | 1 | 87.63 | 86.84 | 92.27 | 92.99 | 75.52 | 76.05 | 83.40 | 85.54 |
|  | 2 | 86.10 | 90.33 | 89.66 | 94.27 | 76.15 | 79.69 | 82.36 | 87.06 |
|  | 3 | 86.63 | 88.59 | 89.17 | 92.38 | 76.00 | 75.38 | 82.12 | 83.08 |
|  | 4 | 83.15 | 88.58 | 87.12 | 94.16 | 70.71 | 77.73 | 77.31 | 86.47 |
|  | 5 | 84.03 | 87.39 | 88.09 | 93.06 | 71.39 | 73.22 | 78.64 | 82.18 |
| 4 | 1 | 81.42 | 79.63 | 89.41 | 90.49 | 68.23 | 67.91 | 80.45 | 82.97 |
|  | 2 | 78.39 | 77.45 | 85.55 | 89.88 | 66.67 | 65.44 | 78.26 | 81.23 |
|  | 3 | 80.02 | 83.02 | 83.60 | 89.46 | 67.83 | 69.22 | 75.92 | 80.19 |
|  | 4 | 84.07 | 84.25 | 91.07 | 92.41 | 69.80 | 68.88 | 80.97 | 80.71 |
|  | 5 | 85.76 | 85.76 | 89.15 | 90.04 | 70.87 | 69.85 | 80.34 | 79.29 |
| 5 | 1 | 84.45 | 94.89 | 85.04 | 95.86 | 70.13 | 80.24 | 72.46 | 83.35 |
|  | 2 | 95.17 | 94.98 | 95.75 | 95.88 | 81.91 | 84.07 | 84.61 | 87.13 |
|  | 3 | 94.66 | 99.96 | 94.67 | 100.00 | 79.93 | 99.49 | 80.58 | 99.99 |
|  | 4 | 94.27 | 94.18 | 94.99 | 97.09 | 80.73 | 80.78 | 84.30 | 86.49 |
|  | 5 | 94.60 | 87.17 | 95.37 | 91.97 | 80.51 | 74.82 | 84.12 | 83.01 |
| 6 | 1 | 67.00 | 63.86 | 75.93 | 74.78 | 54.19 | 49.42 | 68.57 | 64.56 |
|  | 2 | 63.40 | 48.90 | 76.95 | 64.04 | 51.46 | 38.74 | 71.47 | 58.10 |
|  | 3 | 60.77 | 55.13 | 68.86 | 66.39 | 49.10 | 43.80 | 61.19 | 57.69 |
|  | 4 | 67.48 | 61.43 | 78.30 | 80.70 | 56.08 | 51.09 | 71.22 | 74.22 |
|  | 5 | 55.66 | 55.57 | 64.71 | 66.81 | 44.39 | 42.11 | 56.21 | 56.44 |
| 7 | 1 | 89.75 | 86.28 | 93.47 | 94.48 | 74.40 | 72.32 | 82.23 | 85.08 |
|  | 2 | 86.69 | 85.57 | 92.44 | 93.52 | 70.82 | 73.68 | 79.60 | 85.72 |
|  | 3 | 84.07 | 85.27 | 91.40 | 94.48 | 69.97 | 72.06 | 82.00 | 87.03 |
|  | 4 | 85.97 | 87.92 | 92.80 | 95.24 | 70.57 | 73.54 | 81.25 | 85.19 |
|  | 5 | 87.24 | 86.35 | 93.89 | 95.52 | 72.59 | 73.24 | 83.29 | 86.80 |
| 8 | 1 | 93.23 | 92.15 | 96.68 | 96.99 | 80.89 | 82.99 | 87.27 | 90.64 |
|  | 2 | 93.26 | 92.44 | 97.65 | 97.05 | 83.74 | 83.05 | 91.22 | 90.91 |
|  | 3 | 91.05 | 94.39 | 94.48 | 97.98 | 80.18 | 84.38 | 86.08 | 90.67 |
|  | 4 | 91.71 | 92.84 | 96.11 | 97.63 | 80.44 | 80.52 | 88.23 | 88.86 |
|  | 5 | 92.69 | 87.45 | 98.80 | 96.40 | 85.48 | 78.27 | 94.69 | 89.09 |
| 9 | 1 | 80.73 | 59.33 | 83.09 | 63.61 | 73.39 | 54.57 | 80.24 | 63.47 |
|  | 2 | 81.44 | 67.24 | 85.37 | 72.52 | 72.87 | 60.71 | 80.14 | 69.50 |
|  | 3 | 56.98 | 48.40 | 60.57 | 58.15 | 52.11 | 46.78 | 59.57 | 60.17 |
|  | 4 | 64.22 | 66.88 | 66.99 | 69.71 | 57.06 | 60.88 | 62.83 | 67.18 |
|  | 5 | 58.90 | 52.80 | 62.14 | 56.90 | 54.48 | 49.89 | 60.77 | 57.01 |
| 10 | 1 | 88.15 | 80.02 | 92.87 | 85.71 | 75.44 | 67.57 | 83.14 | 76.40 |
|  | 2 | 85.87 | 80.17 | 89.65 | 85.24 | 72.97 | 67.31 | 79.59 | 75.14 |
|  | 3 | 87.18 | 82.94 | 90.69 | 87.10 | 74.22 | 65.53 | 80.58 | 71.86 |
|  | 4 | 89.31 | 80.81 | 93.47 | 86.84 | 76.93 | 68.22 | 83.56 | 77.04 |
|  | 5 | 87.18 | 83.63 | 90.33 | 89.16 | 74.10 | 71.40 | 79.90 | 79.72 |

Figure 1: Per-patient comparison of the scheduled plan and post treatment volumes that received 33 Gy in cubic centimeters.


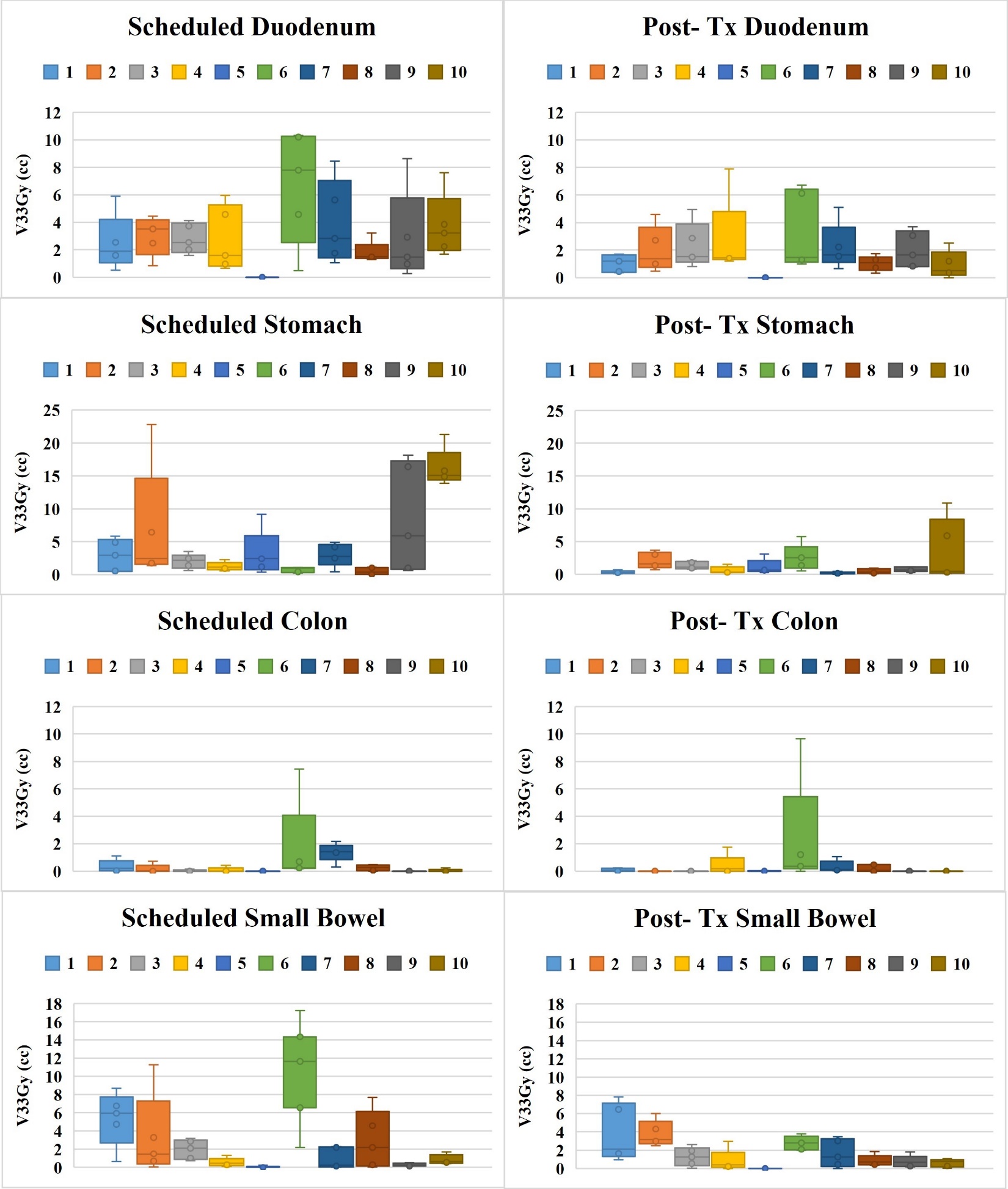

Supplement: Supplementary file 1 [file DataSheet_1.docx]
